# Effect of spironolactone combined with angiotensin-converting enzyme inhibitors and/or angiotensin II receptor blockers on chronic glomerular disease

**DOI:** 10.3892/etm.2013.1335

**Published:** 2013-10-09

**Authors:** WEISONG WANG, LIHUA LI, ZHONGHAI ZHOU, JUNJIE GAO, YINGHAO SUN

**Affiliations:** Department of Nephrology, Cangzhou Central Hospital, Cangzhou, Hebei 061001, P.R. China

**Keywords:** chronic glomerular disease, spironolactone, angiotensin-converting enzyme inhibitor, angiotensin II receptor blocker, aldosterone

## Abstract

The aim of the present study was to observe the effects of spironolactone on urine protein level and kidney function in patients with chronic glomerular disease receiving angiotensin-converting enzyme inhibitors (ACEIs) and/or angiotensin II receptor blockers (ARBs). A total of 221 patients with chronic glomerular disease were divided into spironolactone and control groups. The spironolactone group was treated with spironolactone at a dose of 20 mg/day, in addition to the original treatment regime and doses of ACEIs and/or ARBs. The control group continuously received the original doses of ACEIs and/or ARBs alone. Twenty-four-hour urine protein levels, serum creatinine and potassium, plasma aldosterone (ALD) and blood pressure were monitored at 0, 4, 8, 12 and 16 weeks. The estimated glomerular filtration rates (eGFRs) were calculated based on the obtained serum creatinine results. Following treatment, the urine protein level in the spironolactone group was notably decreased compared with that prior to the treatment, whereas the urine protein level in the control group did not show a significant difference. No significant differences were observed with regard to the renal function, eGFR, serum potassium, plasma ALD and blood pressure in either group prior to and following treatment. In conclusion, spironolactone administration, when co-administered with ACEIs and/or ARBs, markedly decreases the urine protein levels in patients with chronic glomerular disease. The protective effect of spironolactone on renal function remains to be demonstrated.

## Introduction

The renin-angiotensin-aldosterone system (RAAS) is important in the development of chronic kidney disease (CKD). Aldosterone (ALD) in the RAAS affect the vascular wall, leading to fibrosis, glomerular sclerosis and arterial stiffness, which may increase urine protein and promote chronic glomerular disease. This effect is independent of angiotensin II and is not able to be completely blocked by angiotensin-converting enzyme inhibitors (ACEIs) or angiotensin II receptor blockers (ARBs) (1–3). Furthermore, the long-term administration of ACEIs and ARBs has been indicated to result in ALD escape (4–8). These observations have caused clinicians to consider the protective effect of ALD receptor antagonists on renal function for use in patients with CKD, particularly chronic glomerular disease. To date, the majority of studies have focused on the application of spironolactone combined with ACEIs or ARBs in the treatment of diabetic nephropathy (9–12). However, in the current study, the application of spironolactone was extended to various renal glomerular diseases. The curative effects and side-effects of the treatment were then observed.

## Patients and methods

### Clinical data

A total of 221 patients diagnosed with chronic glomerular disease, who received treatment at the Cangzhou Central Hospital (Cangzhou, China) between June 2009 and April 2013, were recruited to the study. Among them, 64 patients had immunoglobulin A (IgA) nephropathy, 65 had membranous nephropathy, 14 had lupus nephritis, 14 had purpura nephritis and 25 had mesangial proliferative nephritis. The diagnoses of the patients, with the exception of 39 patients with clinically confirmed diabetic nephropathy, were confirmed using renal needle biopsy. The inclusion criteria comprised: i) no history of hormone or immunosuppressive agent administration or withdrawal of these drugs for ≥3 months; ii) a history of ACEI and/or ARB treatment for ≥6 months; iii) stable blood pressure <140/90 mmHg; iv) urine protein >0.5 g/24 h; v) plasma albumin >35 g/l; vi) serum creatinine <133 μmol/l; and vii) estimated glomerular filtration rate (eGFR) >30 ml/min/1.73 m^2^. The exclusion criteria included: i) failure to attend further consultation on time; ii) serum potassium >5.0 mmol/l; and iii) side-effects, such as mammoplasia and spargosis.

The enrolled patients were instructed not to eat high-potassium foods and to consume a low-salt diet (sodium chloride intake <6 g/day). When the eGFR of the patients was <60 ml/min/1.73 m^2^, the patients were instructed to consume a low-protein diet (0.8 g/kg/day protein intake). Among the patients, 92 had been treated with benazepril hydrochloride (Lotensin; Beijing Norvatis Pharma Co., Ltd., Beijing, China) at a dosage of 20 mg/day, 73 had been treated with losartan potassium tablets (Cozaar; Hangzhou MSD Pharmaceutical Co., Ltd., Hangzhou, China) at a dosage of 100 mg/day and 56 had been treated with benazepril hydrochloride at a dosage of 10 mg/day in combination with losartan potassium tablets at a dosage of 50 mg/day. No statistically significant differences were observed in the general patient data and primary index baselines prior to the enrollment (Table I).

This study was conducted in accordance with the Declaration of Helsinki and with approval from the Ethics Committee of Cangzhou Central Hospital. Written informed consent was obtained from all participants.

### Methods

The patients were divided into spironolactone and control groups using an open, randomized method. The spironolactone group received spironolactone (Zhejiang Yatai Pharmaceutical Co., Ltd., Shaoxing, China) at a dosage of 20 mg/day, in addition to an ACEI and/or ARB regimen. The control group continued to receive the original dosages of ACEI and/or ARB.

### Observation indices

Twenty-four-hour urine protein levels, serum creatinine and potassium, plasma ALD and blood pressure were monitored 0, 4, 8, 12 and 16 weeks following treatment, respectively. eGFRs were calculated based on the serum creatinine results. The biochemical indices, such as urine protein levels (cerebrospinal fluid/urinary total protein determination kit; SIMES-SIKMA, China), serum creatinine (creatinine determination kit; Sichuan Maker Biotechnology Co., Ltd., Chengdu, China) and serum potassium (ISE internal standard liquid, ISE dilution and ISE reference electrode liquid; Hitachi, Tokyo, Japan), were assessed using a Hitachi 7600 automatic biochemical analyzer (Hitachi), and plasma ALD was analyzed using a Maglumi Automated Chemiluminescence Immunoassay system (Snibe Co., Ltd., Shenzhen, China) and fluorescence immunoassay (using a kit from Snibe Co., Ltd.). The patients’ blood pressure at the right upper extremity was measured three times using a standard cuff mercury sphygmomanometer by the same clinician, between 8.00 and 10.00 a.m., and the mean values were calculated.

### Statistical analysis

All measurement data are presented as the mean ± standard deviation and were compared using a t-test. Enumeration data were analyzed using the χ^2^ test. Analyses were performed using SPSS 11.5 statistical software (SPSS, Inc., Chicago, IL, USA). P<0.05 was considered to indicate a statistically significant difference.

## Results

### Follow-ups

A total of 221 patients were enrolled in this study. Among them, four withdrew from the study during treatment due to severe infection, five failed to attend further consultation on time, one developed mammoplasia and three developed hyperkalemia (serum potassium, 5.8 mmol/l). As a result, 106 patients in the spironolactone group and 102 in the control group completed this study.

### Urine protein levels prior to and following treatment

Following treatment, the urine protein level in the spironolactone group had significantly decreased compared with that prior to treatment (P<0.05), whereas no significant difference was apparent in the control group (P>0.05). At the end-point of treatment, the urine protein level in the spironolactone group was significantly lower than that in the control group (P<0.05). The results are summarized in Table II.

### Renal function prior to and following treatment

Although the serum creatinine levels in the two groups were observed to have increased following treatment compared with those prior to treatment, the differences were not significant (P>0.05). Furthermore, while the eGFRs in the two groups were shown to have decreased following treatment compared with those prior to treatment, the differences were also not significant (P>0.05). In addition, no significant differences were identified in the serum creatinine level in either group prior to and following treatment (P>0.05). The results are summarized in Table III.

### Serum potassium, plasma ALD and blood pressure prior to and following treatment

Neither group demonstrated any significant differences in the level of serum potassium (Table IV), the plasma ALD level (Table V) or systolic or diastolic blood pressure (Table VI) prior to and following treatment (P>0.05).

## Discussion

Glomerulosclerosis is a common pathological condition leading to terminal renal failure that is caused by numerous factors. Changes in glomerular hemodynamics have been suggested to be one of the most important elements in the development of this medical condition. However, in recent years, a number of non-hemodynamic factors have been the subject of particular study, as the understanding of kidney diseases has improved. The abnormal activation of the RAAS is a critical factor leading to the development of chronic glomerular disease. Although numerous studies have demonstrated that ACEIs and/or ARBs exert a curative effect on chronic glomerular disease (13–16), patients receiving the long-term administration of these drugs often re-present with an increase in the plasma ALD level, following a short-term decrease. This phenomenon is known as ‘ALD escape’. The ALD level negatively correlates with the glomerular filtration rate and patients presenting with ALD escape tend to exhibit a more marked aggravation in the urine protein level and renal function, suggesting that the block of the actions of ALD may have a renoprotective effect (17). Therefore, ALD escape provides a theoretical basis for the clinical application of ALD receptor antagonists.

A meta-analysis of 15 studies from Medline, performed by Bomback *et al*(18), showed that ACEIs and/or ARBs combined with mineralocorticoid receptors significantly decreased urine protein levels, rather than affecting renal function, and resulted in severe adverse reactions (18). In this study, we added a low dose of spironolactone to the treatment regimen for patients with chronic glomerular disease who had been treated with ACEIs and/or ARBs for at least six months and whose 24-h urine protein remained between 0.5 and 3.0 g. The results revealed that the urine protein level in the spironolactone group had notably decreased following treatment, whereas that in the control group did not demonstrate a significant change. With regard to the fact that urine protein level is an independent risk factor for the progression of chronic glomerular disease (19–23), spironolactone may be considered to exert a protective effect against chronic glomerular disease.

Spironolactone is a non-selective ALD receptor antagonist which has a chemical constitution similar to that of ALD. Following binding with aldosterone receptors, it influences the formation of ALD-mineralocorticoid receptor complexes, and thereby antagonizes the actions of ALD. Although the mechanisms underlying the effect of spironolactone on urine protein excretion remain unknown, they have been suggested to be correlated with the improvement effects of the drug on renal hemodynamics and non-hemodynamics. Spironolactone has been shown to inhibit the actions of ALD and reduce the expression of inflammatory factors, such as mononuclear macrophage chemoattractant protein, interleukin-6 and interleukin-1β (24), thereby alleviating injuries caused by these factors and protecting renal capillaries. Furthermore, it has been demonstrated that spironolactone reduces the actions of plasma ALD and decreases the stimulatory effect of plasma ALD on transforming growth factor β1 in the plasma and pulmonary tissues. These effects further reduce extracellular-matrix deposition mediated by transforming growth factor β1 and the fibrosis of nephridial tissues and induce the restoration of some sclerotic glomeruli (25,26).

The current study demonstrated that spironolactone, when added to a regimen of ACEIs and/or ARBs, exerted a significant effect on the urine protein level in patients with chronic glomerular disease, but not on blood pressure. Such a marked difference in urine protein did not occur in the control group: Urine protein only decreased by 4.8% by the end-point of treatment (P=0.282). This phenomenon may be attributed to the long-term administration of ACEIs and/or ARBs (14.5±4.8 months) leading to a failure to further significantly decrease the urine protein level. Furthermore, the plasma ALD level in the spironolactone group increased by 8.2%, although no significant difference was observed. The mechanism underlying this phenomenon may be that spironolactone blocks the binding of ALD with its receptors, leaving ALD in a free state. This study was not able to confirm the existence of ALD escape, due to the fact that all the participants had already received treatment with ACEIs and/or ARBs for at least six months and their plasma ALD concentrations were not determined prior to treatment. However, 92 of the 106 patients (~86.8%) showed decreased 24-h urine protein levels. This result indicated that the urine protein-reducing effect of spironolactone in patients with IgA nephropathy was not limited to those with ALD escape, considering that the incidence of ALD escape has been revealed to be ~40% during treatment with ACEIs and/or ARBs (6). Moreover, although serum creatinine levels increased and eGFR decreased in the two groups, no significant differences were observed compared with the values prior to treatment. This phenomenon may be associated with the natural development of renal disease. Therefore, this study was not able to demonstrate the protective effect of spironolactone on renal function, possibly due to the short observation time. To clarify this issue, long-term studies are required in the future.

Spironolactone may lead to hyperkalemia, sexual inadequacy, mammoplasia in males and menstrual disorder and breast tenderness in females (27–30). In this study, three patients developed hyperkalemia. Two of these patients had an eGFR <60 ml/min/1.73 m^2^ and had been eating high-potassium foods, such as oranges and bananas, without following the doctors’ instructions. One patient presented with mammoplasia. The patient had an eGFR of <60 ml/min/1.73 m^2^ and an ALD level that was higher than the average value (9.5 μg/l). Therefore, caution is required with the use of spironolactone for patients at CKD phase III.

In conclusion, ALD influences the expression of a series of cytokines and vasoactive substances by regulating renal vessels and directly acting on cells, thereby causing kidney damage. A low dose of spironolactone, when added to ACEI and/or ARB treatment, notably decreases the urine protein level in patients with chronic glomerular disease. The renoprotective effect remains to be demonstrated using large samples and through long-term observation.

## Figures and Tables

**Table I:** Comparisons of the general data and primary examination indices between groups.

Indices	Spironolactone group (n=106)	Control group (n=102)	P-value
Gender (males:females)	61:45	57:45	0.809
Time^a^ (months)	13.9±4.2	14.5±4.8	0.592
Age (years)	33.7±8.3	34.6±10.2	0.732
Case numbers (ACEI:ARB:ACEI and ARB)	48:32:26	42:36:24	0.727
Urine protein (g/24 h)	1.92±0.71	1.87±0.76	0.936
Serum creatinine (μmol/l)	81.4±22.5	83.6±25.2	0.278
eGFR (ml/min/1.73 m^2^)	65.77±22.21	66.45±24.34	0.523
Serum potassium (mmol/l)	4.23±0.45	4.27±0.41	0.933
Plasma aldosterone (μg/l)	7.64±1.37	7.79±1.39	0.348
Systolic pressure (mmHg)	119.3±13.5	121.1±13.7	0.576
Diastolic pressure (mmHg)	72.2±11.6	68.2±11.1	0.236
Prothrombin time (sec)	11.55±1.33	11.78±1.22	0.478
Partial prothrombin time (sec)	23.08±0.64	23.89±0.69	0.637
Fibrinogen (g/l)	3.07±0.65	3.13±0.63	0.871
Triglyceride (mmol/l)	1.37±0.33	1.39±0.36	0.441
Total cholesterol (mmol/l)	4.91±0.80	4.89±0.97	0.216
Low-density lipoprotein (mmol/)	2.93±0.67	2.91±0.65	0.265

Measurement data are presented as the mean ± standard deviation.

a Duration of angiotensin-converting enzyme inhibitor (ACEI) and/or angiotensin II receptor blocker (ARB) treatment prior to the study. eGFR, estimated glomerular filtration rate.

**Table II:** Comparison of the urine protein levels (g/day) between the groups prior to and following treatment.

Time-point	Spironolactone group	Control group
0 week	1.92±0.71	1.87±0.76
4 weeks	1.79±0.65	1.85±0.69
8 weeks	1.73±0.78	1.81±0.77
12 weeks	1.65±0.83	1.82±0.79
16 weeks	1.59±0.59^a^^,^^b^	1.78±0.81^c^

a P<0.05 compared with the value prior to treatment;

b P<0.05, compared with the control group at 16 weeks;

c P>0.05 compared with the value prior to treatment.

**Table III:** Comparison of the serum creatinine levels and eGFR between groups prior to and following treatment.

	Serum creatinine (μmol/l)		eGFR (ml/min/1.73 m2)	
		
Time-point	Spironolactone group	Control group	Spironolactone group	Control group
0 week	81.4±22.5	83.6±25.2	65.8±22.2	66.5±24.3
4 weeks	82.6±23.4	85.7±24.7	65.1±26.3	65.1±28.9
8 weeks	83.8±21.7	86.4±24.8	64.5±28.4	64.5±33.6
12 weeks	83.2±25.2	87.2±25.6	63.6±26.7	63.9±34.6
16 weeks	84.1±25.7	87.3±27.3	64.1±30.5	63.5±36.9

eGFR, estimated glomerular filtration rate.

**Table IV:** Comparison of the plasma potassium levels between groups prior to and following treatment.

	Serum potassium level (mmol/l)	
	
Time-point	Spironolactone group	Control group
0 week	4.23±0.45	4.27±0.41
4 weeks	4.15±0.43	4.18±0.36
8 weeks	4.23±0.49	4.23±0.44
12 weeks	4.36±0.41	4.30±0.39
16 weeks	4.38±0.44	4.38±0.43

**Table V:** Comparison of the plasma aldosterone levels between groups prior to and following treatment.

	Plasma aldosterone level (μg/l)	
	
Time-point	Spironolactone group	Control group
0 week	7.64±1.37	7.79±1.39
4 weeks	8.26±1.35	7.83±1.17
8 weeks	8.33±1.29	7.85±1.27
12 weeks	8.27±1.27	7.99±1.04
16 weeks	8.31±1.43	7.95±0.96

**Table VI:** Comparison of the blood pressure (mmHg) between groups prior to and following treatment.

	Spironolactone group		Control group	
		
Time-point	SBP (mmHg)	DBP (mmHg)	SBP (mmHg)	DBP (mmHg)
0 week	119.3±13.5	72.2±11.6	121.1±13.7	68.2±11.1
4 weeks	118.6±11.9	71.9±9.9	119.3±12.7	66.5±11.7
8 weeks	118.9±13.3	72.5±10.6	119.9±13.9	65.9±12.2
12 weeks	118.1±12.7	72.9±10.8	119.5±14.3	65.4±11.9
16 weeks	117.6±12.7	70.4±10.1	120.9±14.8	65±12.3

SBP, systolic blood pressure; DBP, diastolic blood pressure.
